# Progesterone promotes maternal–fetal tolerance by reducing human maternal T‐cell polyfunctionality and inducing a specific cytokine profile

**DOI:** 10.1002/eji.201445404

**Published:** 2015-08-28

**Authors:** David Lissauer, Suzy A. Eldershaw, Charlotte F. Inman, Aravinthan Coomarasamy, Paul A. H. Moss, Mark D. Kilby

**Affiliations:** ^1^ Centre for Women's and Children's HealthCollege of Medical and Dental SciencesUniversity of BirminghamBirminghamUK; ^2^ School of Cancer SciencesCollege of Medical and Dental SciencesUniversity of BirminghamBirminghamUK

**Keywords:** Human, IL‐4, Maternal–fetal tolerance, Progesterone, T cell

## Abstract

Progesterone is a steroid hormone essential for the maintenance of human pregnancy, and its actions are thought to include promoting maternal immune tolerance of the semiallogenic fetus. We report that exposure of maternal T cells to progesterone at physiological doses induced a unique skewing of the cytokine production profile of CD4^+^ and CD8^+^ T cells, with reductions not only in potentially deleterious IFN‐γ and TNF‐α production but also in IL‐10 and IL‐5. Conversely, production of IL‐4 was increased. Maternal T cells also became less polyfunctional, focussing cytokine production toward profiles including IL‐4. This was accompanied by reduced T‐cell proliferation. Using fetal and viral antigen‐specific CD8^+^ T‐cell clones, we confirmed that this as a direct, nonantigen‐specific effect. Yet human T cells lacked conventional nuclear progesterone receptors, implicating a membrane progesterone receptor. CD4^+^ and CD8^+^ T cells responded to progesterone in a dose‐dependent manner, with subtle effects at concentrations comparable to those in maternal blood, but profound effects at concentrations similar to those at the maternal–fetal interface. This characterization of how progesterone modulates T‐cell function is important in understanding the normal biology of pregnancy and informing the rational use of progesterone therapy in pregnancies at risk of fetal loss.

## Introduction

During human pregnancy, the maternal immune system is faced with the challenge of establishing ongoing tolerance to the semiallogenic fetus, while retaining the ability to mount a robust cellular immune response to pathogens.

Recognition of fetal antigens by the maternal immune system occurs at the decidual–trophoblast interface, and peripherally due to the shedding of fetal antigens into the maternal circulation 1. This leads to maternal immunological awareness of the developing fetus 2. Indeed, a functional maternal CD8^+^ T‐cell immune response to fetal antigens has been demonstrated during 3 and after 4 human pregnancy. Despite pregnancy‐induced activation of maternal T cells 5, the fetus is protected by a network of immunoregulatory mechanisms. These mechanisms include the induction of conceptus‐specific induced TReg cells 6, chemokine gene silencing (that prevents the accumulation of activated T cells) 7, restricted presentation of fetal antigens 8, and skewing of maternal T‐cell cytokine production toward a more “tolerogenic profile” 9.

Progesterone (P4, Pregn‐4‐ene‐3,20‐dione) is a naturally occurring steroid hormone, produced by the placenta during human pregnancy. This hormone increases in concentration in maternal peripheral blood as pregnancy progresses, and is found in far higher concentrations in placental tissue (<1 μM third trimester serum, 10 μM in placenta) 10, 11, 12. The essential role it plays in the maintenance of pregnancy is demonstrated as progesterone receptor (PR) antagonism with mifepristone (RU486) results in the cessation of pregnancy. Furthermore, progesterone is also used therapeutically in pregnancy to reduce the risk of preterm delivery 13 and improve outcomes in women with recurrent unexplained miscarriages 14, though these clinical uses remain the subject of ongoing clinical trials to demonstrate efficacy and safety.

Conventionally, progesterone's actions to maintain pregnancy have been thought related to myometrial quiescence 15 but it may also have immunomodulatory properties important in maternal tolerance of the fetus.

The potential immunomodulatory effects of progesterone are varied 16 but specific effects on T‐cell function have been described. In murine models, it has been demonstrated that the nuclear PR is present in CD4^+^ T cells and progesterone's action via this receptor reduces CD4^+^ T‐cell effector activity, by transcriptional repression of the IFN‐γ gene 17. Progesterone has also been demonstrated to promote generation of murine induced TReg cells and this is mediated by nuclear PR interactions 18.

Progesterone induces a reduction in the peripheral blood Th1/Th2 ratio. Raghupathy et al. demonstrated progesterone‐induced modulation of cytokine production by mitogen‐stimulated PBMCs from women with unexplained RM and observed a significant reduction in IFN‐γ and TNF‐α together with increased production of IL‐4. However, the lymphocytes responsible for this effect were not determined and the immunological effects were not fully characterized 11.

Evidence is now accumulating that progesterone can also induce rapid, nongenomic responses in T cells 19 and that these are effects independent of glucocorticoid signaling 20 but the mechanisms behind these and the immunological consequences remain poorly defined. The classical PRs are nuclear (PR) with two isoforms A and B. However, in contrast to the mouse, most studies have not identified these receptors in human T cells [21]. More recently, attention has turned to two families of membrane PRs, belonging to the progestin and adipoQ receptor (PAQR) family (also known as membrane progestin receptors (mPRs)) and the progesterone receptor membrane component (PGRMC) receptors), suggested as potential mediators of progesterone effects on T‐cell function. In humans, there are three members of the PAQR family (PAQR5, PAQR7, and PAQR8) and two members of the PGRMC family (PGRMC1 and PGRMC2) 21.

In this study, we have characterized in detail the dose‐dependent effects of progesterone on cytokine production of maternal and control CD4^+^ and CD8^+^ T cells. In addition, we have studied its effects on fetal (HY) antigen specific T‐cell clones, isolated from pregnancy, interacting with their cognate antigen and compared this to antiviral T‐cell controls. Multiparameter flow cytometry provides a far clearer understanding of the effects of progesterone on cytokine production, polyfunctionality, and proliferation of maternal CD4^+^ and CD8^+^ T cells. These new insights have important consequences for our understanding of the biology of pregnancy.

## Results

### Progesterone modulates the pattern of T‐cell cytokine production in a dose‐dependent manner

**Figure 1:**
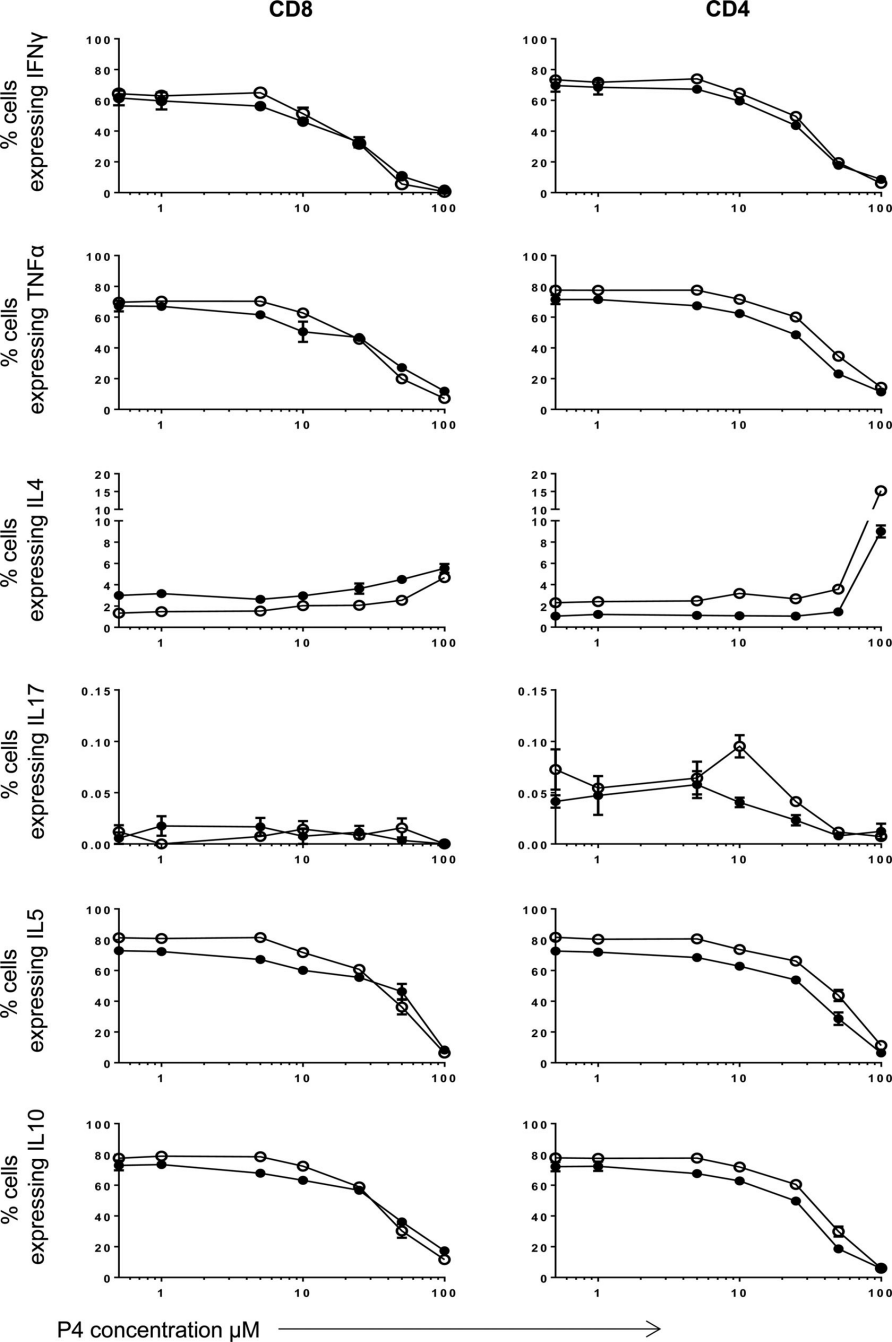
The effect of progesterone on cytokine production by human maternal CD8^+^ and CD4^+^ T cells is dose‐dependent. PBMCs from healthy maternal (●) and control (○) donors (*n* = 1, experiment in triplicate) were treated with PHA and increasing concentrations of progesterone (P4) from 0.5 to 100 μM. The effect of progesterone treatment on the production of IFN‐γ, TNF‐α, IL‐4, IL‐17, IL‐5, and IL‐10 was measured by flow cytometry. Data are shown as mean + SEM from a single experiment.

In order to determine the potential ability of progesterone to influence cytokine production by T cells, we initially stimulated T cells with mitogen for 48 h in the presence of varying concentrations of progesterone (0–100 μM). Cytokine production was measured in PBMCs isolated from maternal and control donors and the percentage of CD8^+^ and CD4^+^ T cells expressing IFN‐γ, TNF‐α, IL‐5, and IL‐10 was determined using flow cytometry (Fig. 1). Incubation with increasing concentrations of progesterone led to a reduction in the production of all four cytokines by both T‐cell subsets, with modest suppression observed at concentrations below 10 μM, and a dramatic and near complete attenuation of cytokine production at levels up to 100 μM. Remarkably, the percentage of cells expressing IL‐4 demonstrated the opposite pattern, with an increase in cells expressing this cytokine, also in a dose‐responsive pattern. The percentage of maternal CD4^+^ T cells, which expressed IL4, increased from a mean of 1.03–9% at the highest progesterone concentration. A similar pattern was observed in maternal CD8^+^ T cells. The proportion of CD4^+^ and CD8^+^ T cells, which produced IL‐17, was low and no clear influence of progesterone was observed.

The pattern of cytokine production within CD8^+^ and CD4^+^ T cells was comparable between maternal and control cells (Fig. 1). Suppression of IFN‐γ, TNF‐α, IL‐5, and IL‐10 appeared to start at a slightly lower progesterone concentration in maternal cells compared to the controls, warranting more detailed examination of this. Based on this initial data, and taking into account physiological levels of progesterone during pregnancy, we selected progesterone concentrations of 1 and 10 μM as a basis for detailed studies on T‐cell function.

### Progesterone reduces IFN‐γ, TNF‐α, IL‐5, and IL‐10 and increases IL‐4 production by CD8^+^ T cells

**Figure 2:**
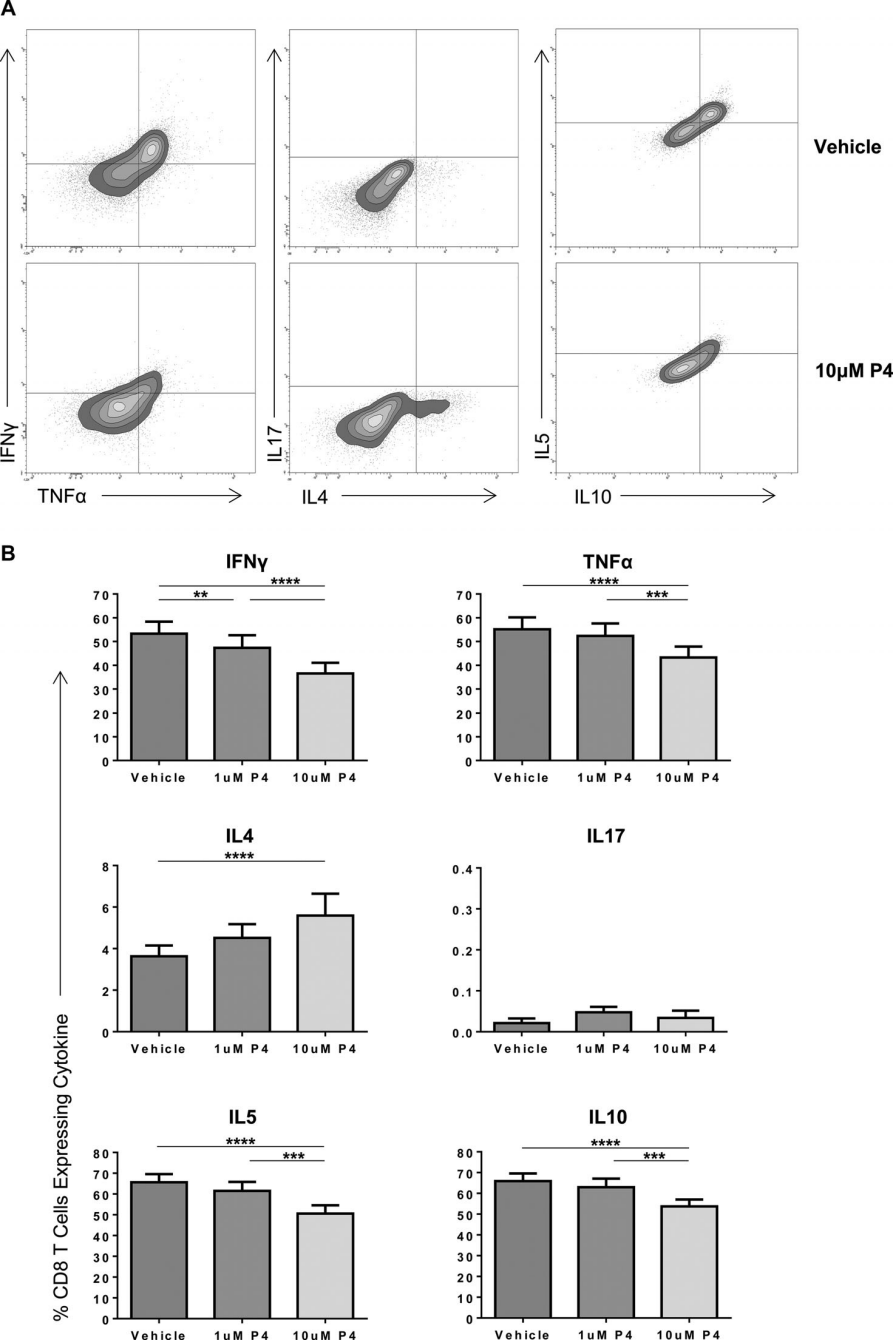
Treatment of maternal PBMCs with physiological concentrations of progesterone alters the cytokine expression of CD8^+^ T cells. PBMCs from healthy maternal donors were treated with PHA and either DMSO (vehicle), or 1 or 10 μM progesterone. The effect of progesterone treatment on production of IFN‐γ, TNF‐α, IL‐4, IL‐17, IL‐5, and IL‐10 was measured by flow cytometry. (A) A representative flow plot of PBMCs from one patient treated with DMSO and 10 μM progesterone (P4) is shown. (B) The cytokine expression of maternal CD8^+^ T cells overall when treated with different progesterone concentrations or vehicles is shown as mean + SEM of 13 donors. *p ≤ 0.05, **p ≤ 0.01, ***p ≤ 0.001, ****p ≤ 0.0001, one‐way ANOVA, repeated measures, and Bonferroni multiple comparison.

The effect of incubation with 1 or 10 μM progesterone on the cytokine profile of activated CD8^+^ T cells from a range of maternal donors (*n* = 13) was assessed by flow cytometry (Fig. 2A; additional gating strategy shown in Supporting Information Fig. 1). Overall, compared to treatment with vehicle control, treatment with 10 μM progesterone resulted in a significant decrease in the mean percentage of CD8^+^ T cells expressing IFN‐γ (53.3 vs. 36.6%, *p* < 0.0001), TNF‐α (55.2 vs. 43.3%, *p* < 0.0001), IL‐5 (65.6 vs. 50.6%, *p* < 0.0001), and IL‐10 (65.9 vs. 53.7%, *p* < 0.0001; Fig. 2B). Exposure to 1 μM progesterone also produced a significant reduction in the percentage of CD8^+^ T cells expressing IFN‐γ (53.3 vs. 47.3%, *p* < 0.01; Fig. 2B) although the influence on the other cytokines was less marked.

Interestingly, treatment with 10 μM progesterone significantly increased the percentage of CD8^+^ T cells expressing the Th2 cytokine IL‐4 compared to vehicle control (3.6 vs. 5.6%, *p* < 0.05; Fig. 2B). No significant changes in the percentage of these lymphocytes expressing IL‐17 was observed, with very low percentages of cells expressing this cytokine.

### Progesterone reduces IFN‐γ, TNF‐α, IL‐5, and IL‐10 and increases IL‐4 production by CD4^+^ T cells

**Figure 3:**
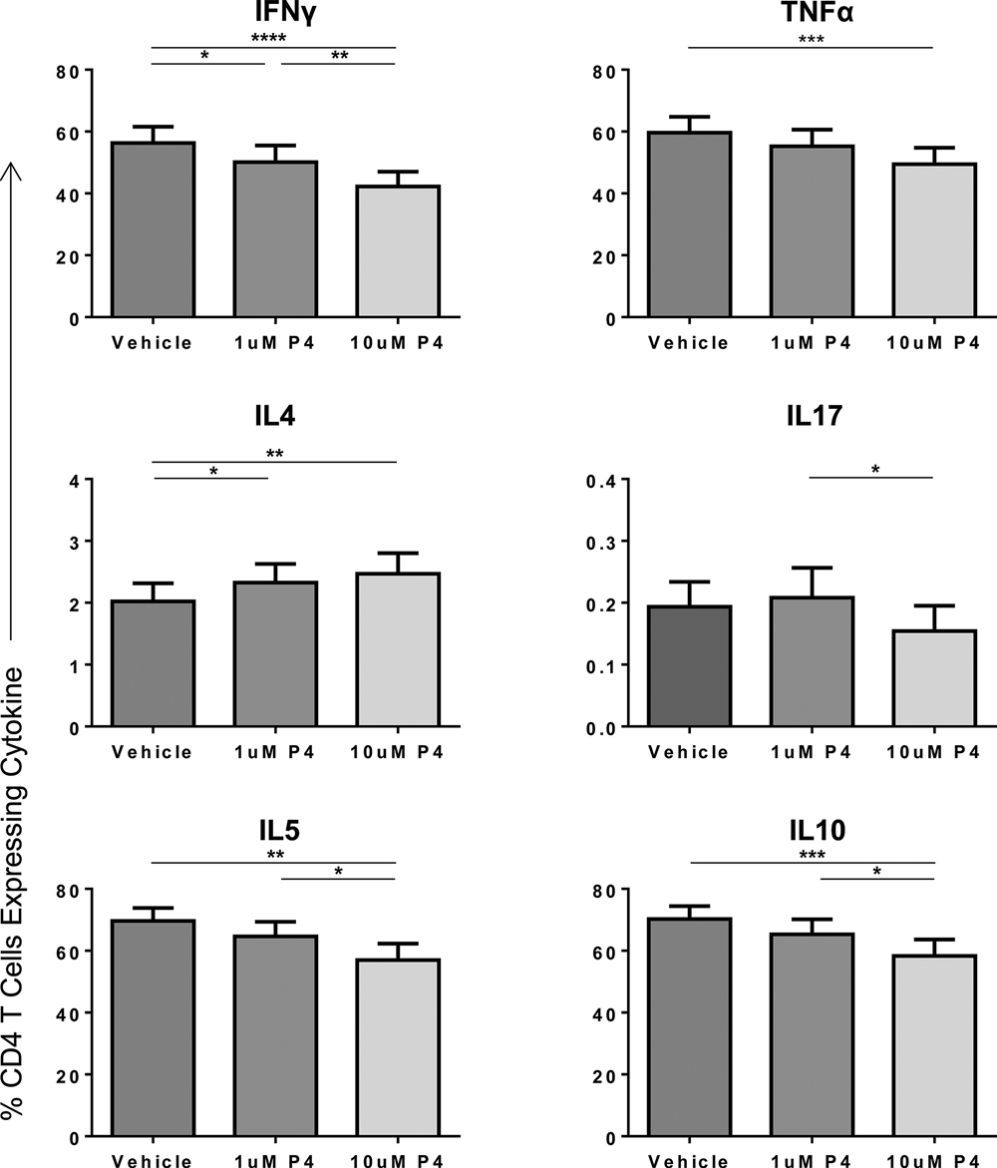
Treatment of maternal PBMCs with physiological concentrations of progesterone alters the cytokine profile of CD4^+^ T cells. PBMCs from maternal donors were treated with PHA and either DMSO (vehicle), or 1 or 10 μM progesterone and the effect of progesterone treatment on production of IFN‐γ, TNF‐α, IL‐4, IL‐17, IL‐5, and IL‐10 was measured by flow cytometry. The cytokine profile of maternal CD4^+^ T cells overall when treated with different progesterone concentrations or vehicle is shown as mean + SEM of 13 donors. *p ≤ 0.05, **p ≤ 0.01, ***p ≤ 0.001, ****p ≤ 0.0001, one‐way ANOVA, repeated measures, and Bonferroni multiple comparison test.

The effect of progesterone on cytokine production by CD4^+^ T cells was also examined in maternal donors (*n* = 13; Fig. 3). The influence of progesterone on cytokine production from CD4^+^ T cells was comparable to that seen for CD8^+^ cells although effects were somewhat more marked. Treatment of PBMCs with 10 μM progesterone resulted in a decrease in the percentage of CD4^+^ T cells expressing IFN‐γ (56.3% down to 42.3%, *p* < 0.0001). Comparable reductions were also observed for production of TNF‐α (59.6 vs. 49.4%, *p* < 0.001), IL‐5 (69.7 vs. 57.0%, *p* < 0.01), and IL‐10 (70.2 vs. 58.3%, *p* < 0.001; Fig. 3). Compared to treatment with the vehicle control, 1 μM progesterone also demonstrated a significant reduction in the percentage of CD4^+^ T cells expressing IFN‐γ (56.3 vs. 50.1%, *p* < 0.05). As seen with CD8^+^ T cells, the percentage of CD4^+^ cells, which expressed IL‐17, was low although a small further decrease was observed when cells were treated with 10 μM progesterone compared to 1 μM progesterone (0.21 vs. 0.15%, *p* < 0.05).

Overall, the effects of progesterone on IL‐4 production were greater than those observed in CD8^+^ T cells, with a significant increase observed in the proportion of cells that produced IL‐4 at concentrations of both 1 μM (2.0–2.3%, *p* < 0.05) and 10 μM (2.0–2.5%, *p* < 0.01).

### Progesterone treatment increases the percentage of IL‐4‐expressing maternal CD8^+^ T cells

The initial dose–response experiment (Fig. 1) suggested progesterone‐mediated attenuation of IFN‐γ, TNF‐α, IL‐5, and IL‐10 may be greater for maternal T cells than controls. IL‐4 production by maternal CD8^+^ T cells appeared to be greater compared to controls. However, due to the limited number of subjects in this initial experiment, we further examined this question using a larger patient cohort.

**Figure 4:**
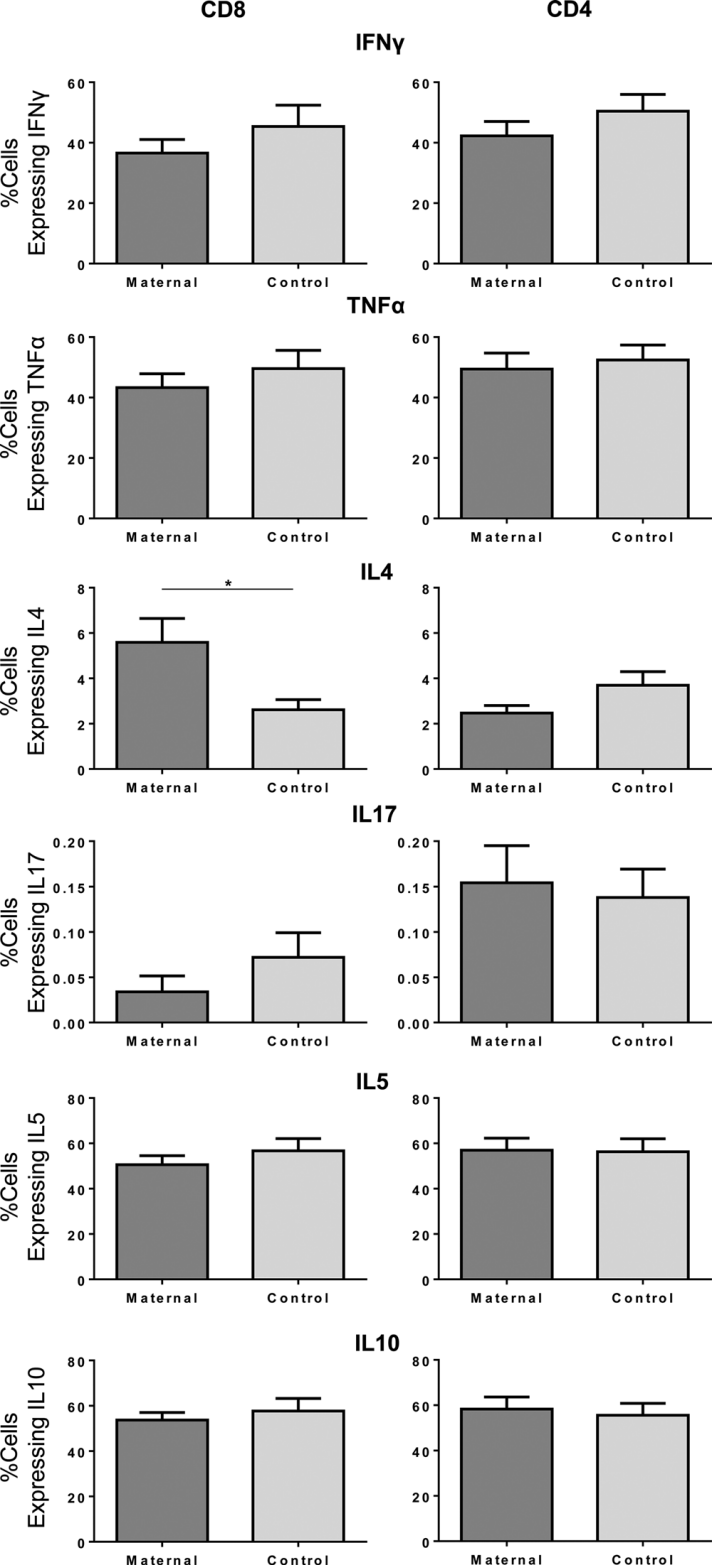
The percentage of CD8^+^ T cells expressing IL‐4 is significantly higher in maternal subjects compared with that of controls following treatment with 10 μM progesterone. The cytokine profile of maternal CD4^+^ and CD8^+^ T cells (dark gray, *n* = 13) was compared with that of control cells (light gray, *n* = 11) where PBMCs were treated with PHA and 10 μM progesterone, as measured by flow cytometry. Data shown as mean + SEM of indicated numbers of donors. *p ≤ 0.05, unpaired *t*‐test.

The percentage of cells expressing cytokines following treatment with 10 μM progesterone was compared between maternal and control samples (Fig. 4). At this progesterone concentration, the percentage of maternal CD8^+^ T cells expressing IL‐4 was significantly higher than the controls (5.6 vs. 2.6%, *p* < 0.05) but there appeared to be no difference in the percentage of either CD8^+^ or CD4^+^ T cells expressing IFN‐γ, TNF‐α, IL‐17, IL‐5, and IL‐10 by maternal and control cells, or CD4^+^ T cells expressing IL‐4.

### Treatment of PBMCs with progesterone reduces the polyfunctionality of CD8+ and CD4+ T cells

**Figure 5:**
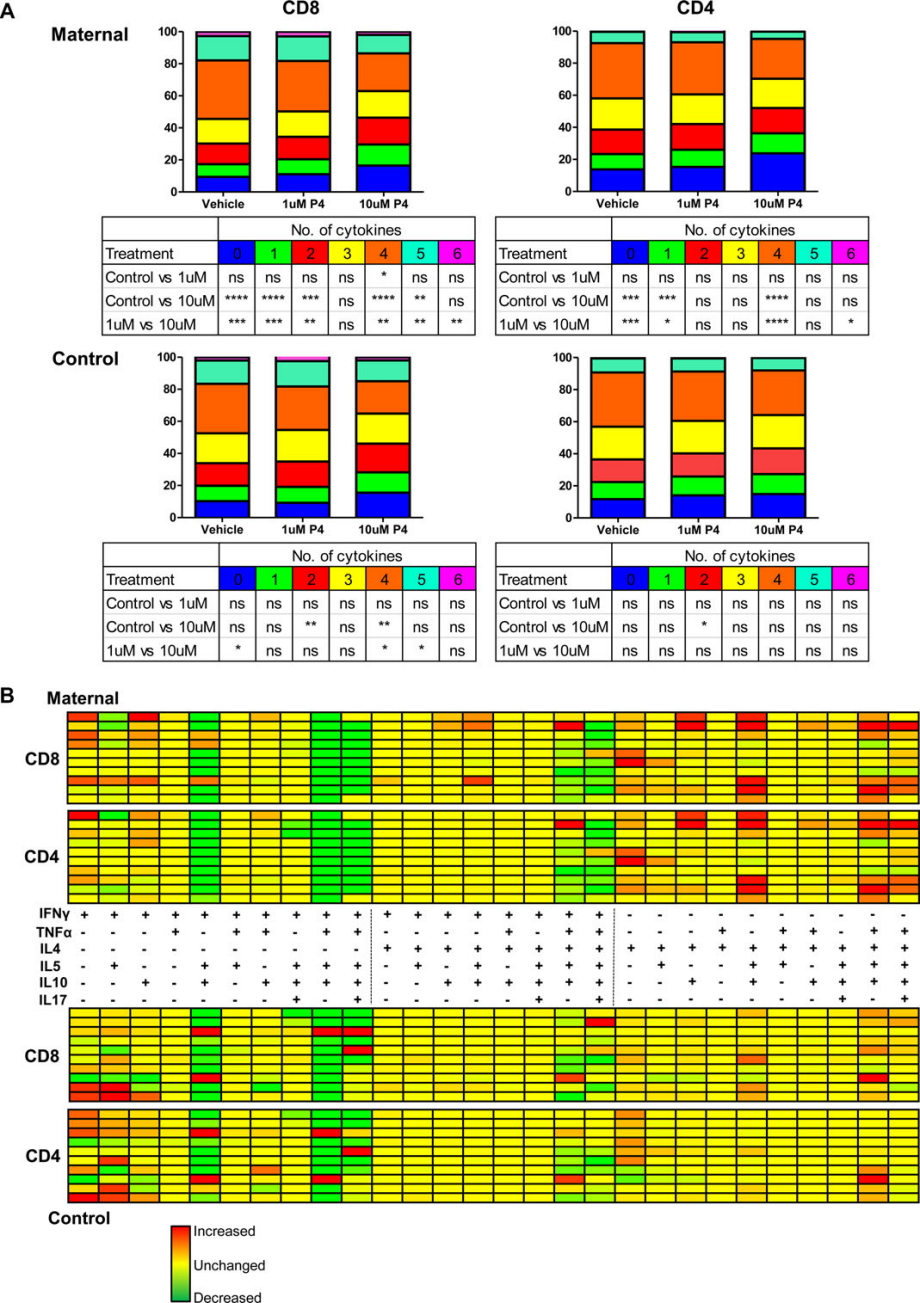
Progesterone reduces the polyfunctional cytokine profile of CD8^+^ and CD4^+^ maternal T cells and favors a more IL‐4 dominant profile. (A) A Boolean gating strategy was established to look at the percentage of CD8^+^ and CD4^+^ T cells expressing from zero to six cytokines from both the maternal (top) and control (bottom) donors (*n* = 10 of each group), where PBMCs had been treated with PHA and either DMSO (vehicle), or 1 or 10 μM progesterone. *p ≤ 0.05, **p ≤ 0.01, ***p ≤ 0.001, ****p ≤ 0.0001, one‐way ANOVA, repeated measures, and Bonferroni multiple comparison test. (B) The same strategy was then used to look at the percentage of CD8^+^ and CD4^+^ T cells expressing each individual cytokine and every possible combination up to six cytokines. The differential absolute expression between the vehicle and 10 μM progesterone was used to create a heat map indicating the change in cytokine combinations when maternal or male PBMCs were treated with PHA and 10 μM progesterone. The change in cytokine expression following progesterone treatment is represented by a range of colors of varying green (increase) and red (decrease) intensity, denoting the percentage change in cytokine production with progesterone treatment. The map has been laid out in three sections; the left section is combination of cytokines including IFN‐γ but excluding IL‐4, the middle section is combination including both IFN‐γ and IL‐4, and the right section is inclusive of IL‐4 but excludes IFN‐γ.

Using single‐tube multicolor flow cytometry, we went on to determine the polyfunctional properties of CD8^+^ and CD4^+^ T cells by examining the simultaneous expression of up to six cytokines within individual cells. This pattern was determined after stimulation with mitogen, and in the presence or absence of progesterone at either 1 or 10 μM (Fig. 5A) with a Boolean gating strategy examining the expression of all cytokine combinations. Treatment of maternal PBMCs with 10 μM progesterone significantly decreased the polyfunctional cytokine production profile of both CD4^+^ and CD8^+^ T‐cell subsets. The percentage of CD8^+^ T cells expressing four cytokines fell from 36.6 to 23.5% (*p* < 0.0001), and the proportion capable of simultaneous production of five cytokines fell from 15.2 to 11.5% (*p* < 0.01). Conversely, the percentage of CD8^+^ T cells expressing zero, one, and two cytokines was significantly increased (9.4 vs. 16.5%, 7.9 vs. 13.4%, *p* < 0.0001, and 12.9 vs. 16.8%, *p* < 0.001, respectively) (Fig. 5A, upper left panel). A similar pattern was observed in maternal CD4 T cells (Fig. 5B, upper right panel). Thus, progesterone treatment had a significant effect on reducing the polyfunctional cytokine profile of maternal CD8^+^ and CD4^+^ T cells.

The effect of progesterone on the number of cytokines expressed by CD4^+^ and CD8^+^ T cells from the control cohort appears to be less dramatic, and the effects were seen predominantly in CD8^+^ rather than CD4^+^ T cells (Fig. 5A, lower panel). A significant decrease was observed in the percentage of CD8^+^ T cells that expressed four cytokines following treatment with 10 μM progesterone (30.9 vs. 20.2%, *p* < 0.01), as well as an increase in the percentage expressing two cytokines (14.0 vs. 17.9%, *p* < 0.01; Fig. 5A, lower left panel). In comparison, there was only a small significant increase in the percentage of CD4^+^ T cells expressing two cytokines upon treatment with 10 μM progesterone compared to vehicle (16.1 vs. 14.0%, *p* < 0.05; Fig. 5A, lower right panel).

Overall, progesterone had a marked effect on reducing the polyfunctional cytokine potential of CD4^+^ and CD8^+^ T cells, and this appeared more pronounced on maternal T cells compared to controls.

### Progesterone treatment increases production of cytokine combinations that include IL‐4

Using the Boolean gating strategy, we were also able to determine the composition of the cytokines produced by each of the T cells simultaneously. As we previously observed a clear increase in T cells producing IL‐4 following incubation with progesterone, we went on to examine the relative contribution of IL‐4 within the total polyfunctional cytokine production profile of cells following progesterone treatment. The percentage of CD8^+^ and CD4^+^ T cells expressing each combination of cytokines was determined and results were represented as a heat map (Fig. 5B) from both the maternal (upper) and control (lower) cohorts. The absolute change in the percentage of cells that express each cytokine combination was determined, relative to vehicle control, following exposure to 10 μM progesterone.

The heat map demonstrates a clear and consistent pattern between individuals in each group. For maternal CD8^+^ and CD4^+^ T cells, progesterone treatment induces an increased expression of cytokine combinations where IL‐4 is expressed in the absence of IFN‐γ. Conversely for patients from the control cohort, there is little change in this feature in response to progesterone.

It is also notable that the percentage of T cells expressing IFN‐γ alone increases for both maternal and control cells. This is because although the percentage of cells expressing IFN‐γ is reduced overall (Figs. 2, 3, 4), the reduction in polyfunctionality (Fig. 5) means that those cells that are expressing IFN‐γ are doing so with a restricted numbers of other cytokines, hence this particular combination is becoming more prevalent with progesterone treatment.

### Progesterone treatment reduces T‐cell proliferation

**Figure 6:**
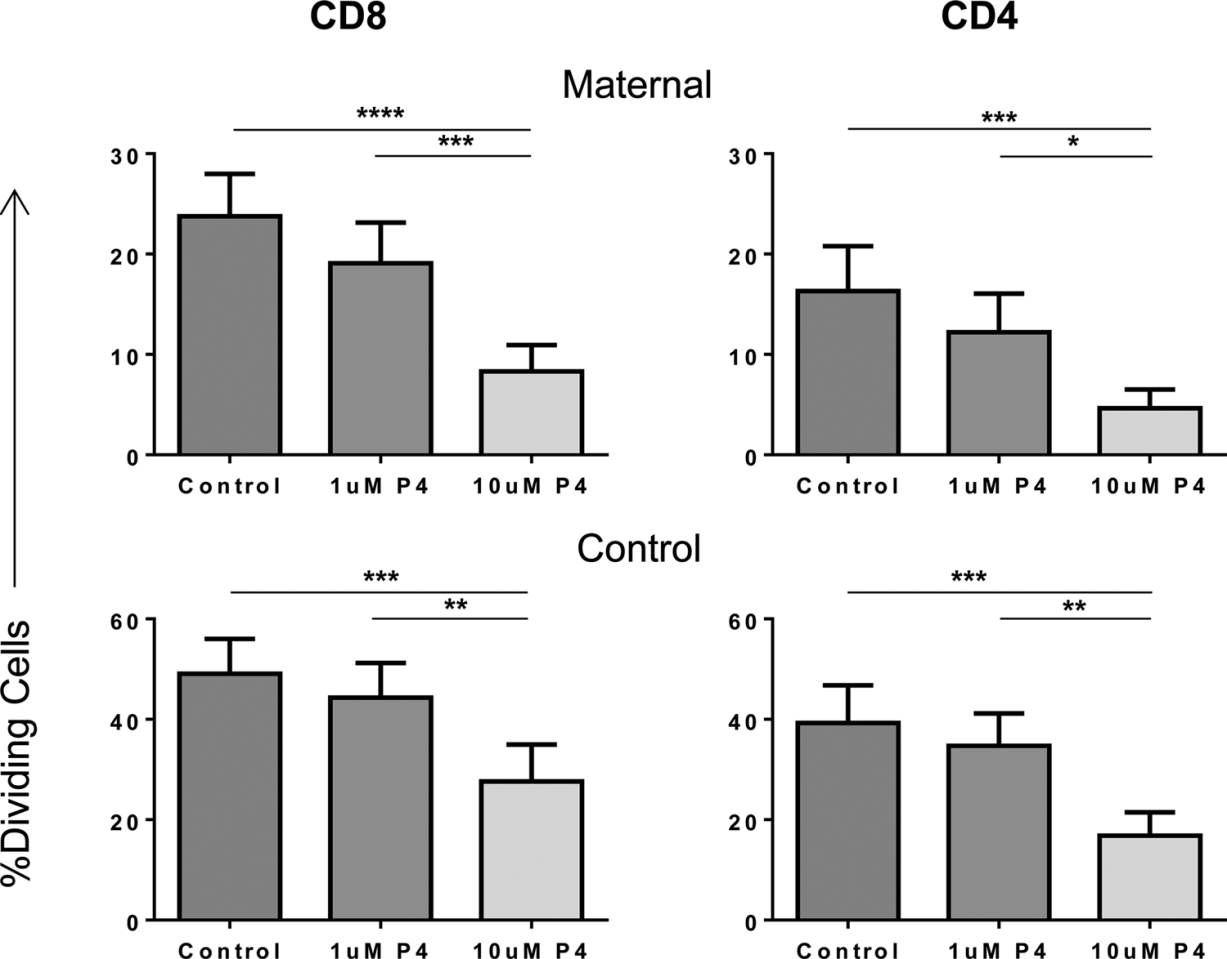
Progesterone treatment reduces the proliferation of CD8^+^ and CD4^+^ T cells. PBMCs from maternal and control donors were labeled with CellTrace™, treated with PHA and either vehicle, or 1 or 10 μM progesterone. The percentages of dividing maternal and control CD8^+^ and CD4+ T cells overall when treated with different progesterone concentrations or vehicle were then measured by flow cytometry and shown as mean + SEM of five donors in each group. *p ≤ 0.05, **p ≤ 0.01, ***p ≤ 0.001, ****p ≤ 0.0001, one‐way ANOVA, repeated measures, and Bonferroni multiple comparison test.

The influence of progesterone on the proliferative potential of CD8^+^ and CD4^+^ T cells, following stimulation with mitogen, was examined (Fig. 6). Incubation with progesterone led to a marked reduction in the percentage of cells that underwent proliferation in both maternal and control CD8^+^ and CD4^+^ T cells. Specifically, the addition of 10 μM progesterone led to a reduction in the proportion of maternal cells that underwent proliferation within 48 h from 23.6 to 8.3% (*p* < 0.0001) in CD8^+^ and 16.3 to 4.6% (*p* < 0.001) in CD4^+^ T cells. In the control cohort, a similar phenomenon was observed with a significant reduction in proliferation of both CD8^+^ (*p* < 0.001) and CD4^+^ (*p* < 0.001) T cells.

### Progesterone attenuates IFN‐γ production by antigen‐specific activation of CD8^+^ T cells

Next we assessed the potential influence of progesterone on the function of antigen‐specific CD8^+^ T‐cell clones. This work was performed to address three specific questions. First, to determine if progesterone was able to modulate the pattern of T‐cell responses following antigen‐specific rather than mitogen activation. Second, to assess if the actions of progesterone could be demonstrated as a direct effect on isolated T cells, thereby excluding the potential for indirect effects when it is added to mixed PBMCs. Finally, to examine if there may be differential responses to progesterone in CD8^+^ T‐cell clones specific to fetal antigens isolated from women during pregnancy compared to clones that have specificity for viral antigens.

CD8^+^ T‐cell clones specific for the HY‐derived minor histocompatibility antigen FIDSYICQV restricted by HLA*0201 were activated by exposure to irradiated HLA*0201 B‐LCL loaded with either the HY peptide or an irrelevant peptide. The production of IFN‐γ and IL‐4 in response to stimulation was detected by intracellular cytokine staining, and the influence of progesterone on the pattern of cytokine response was determined. The specificity of the T‐cell clones was confirmed by MHC‐peptide multimer staining (Supporting Information Fig. 2).

**Figure 7:**
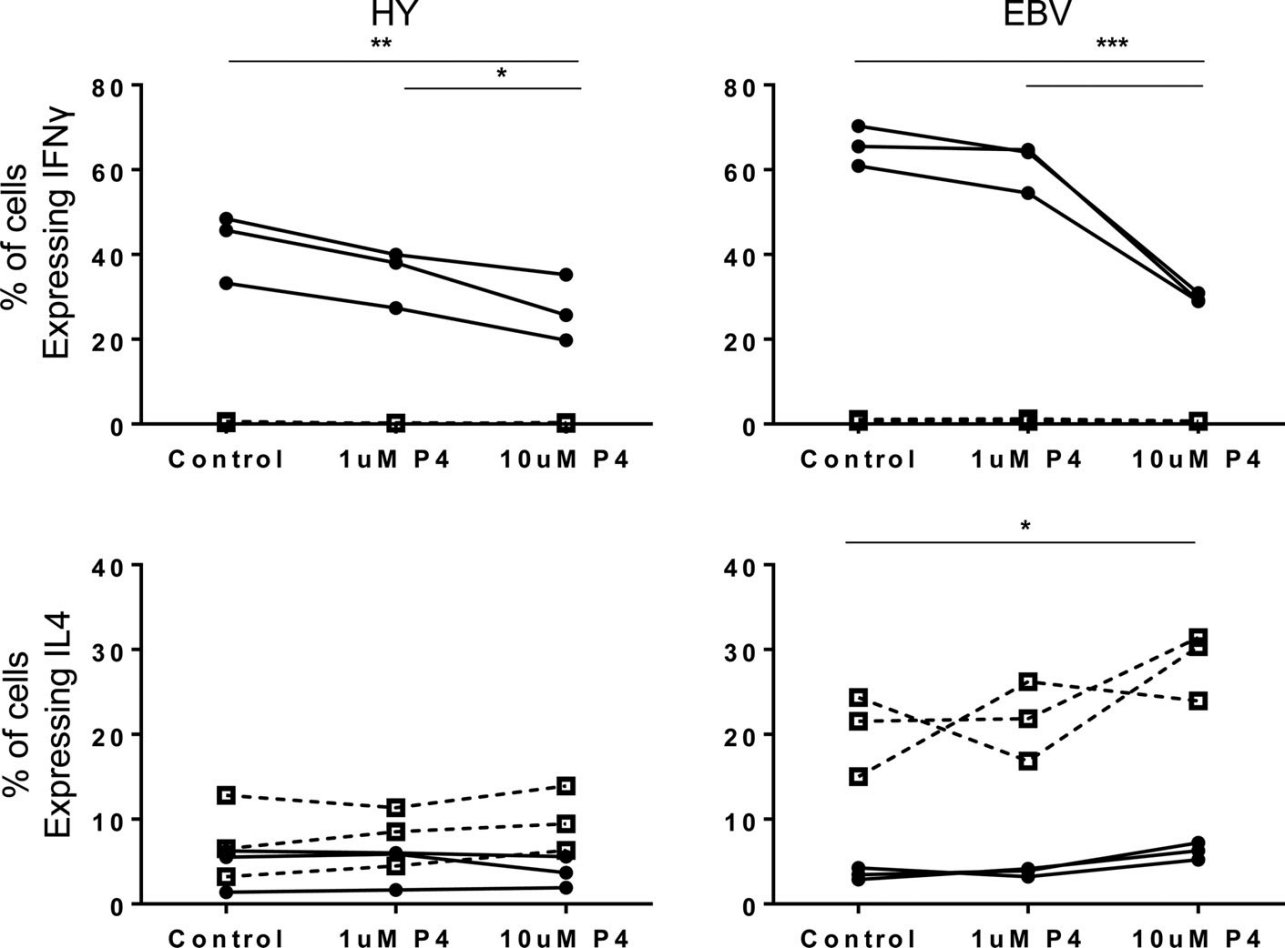
Treatment of antigen‐specific CD8^+^ T‐cell clones with progesterone suggests that changes in the cytokine profile of these cells occur through both direct and indirect effects. Overall effects of progesterone treatment on the percentage of HY antigen‐specific CD8^+^ T cells expressing IFN‐γ (top) and IL‐4 (bottom) following incubation with target cells loaded with HY‐specific peptide (●) or irrelevant peptide (□) (left) and on EBV clones specific for the GLC peptide incubated with target cells loaded with GLC peptide (●) or knock out cells unable to express GLC peptide (□) (right). Three independent HY clones, and three independent EBV clones were tested over three experiments; *p ≤ 0.05, **p ≤ 0.01, ***p ≤ 0.001, ****p ≤ 0.0001, one‐way ANOVA, repeated measures, and Bonferroni multiple comparison test.

The percentage of cells within the CD8^+^ T‐cell clones, which produced IFN‐γ after stimulation with antigen, was markedly downregulated by 10 μM progesterone, falling from a mean of 42.48% to a mean of 26.91% (*p* = 0.01) (Fig. 7) . A similar effect was observed previously in CD8^+^ T cells, irrespective of their origin from maternal or control PBMCs (Fig. 2, 3, 4). Importantly, a similar effect was observed in response to fetal or viral antigen target. This demonstrates that progesterone‐induced suppression of IFN‐γ production following antigen‐specific stimulation is not induced solely against T‐cell clones that are primed during pregnancy.

### mPRs but not nuclear PRs are detectable on CD8^+^ and CD4^+^ T cells

**Figure 8:**
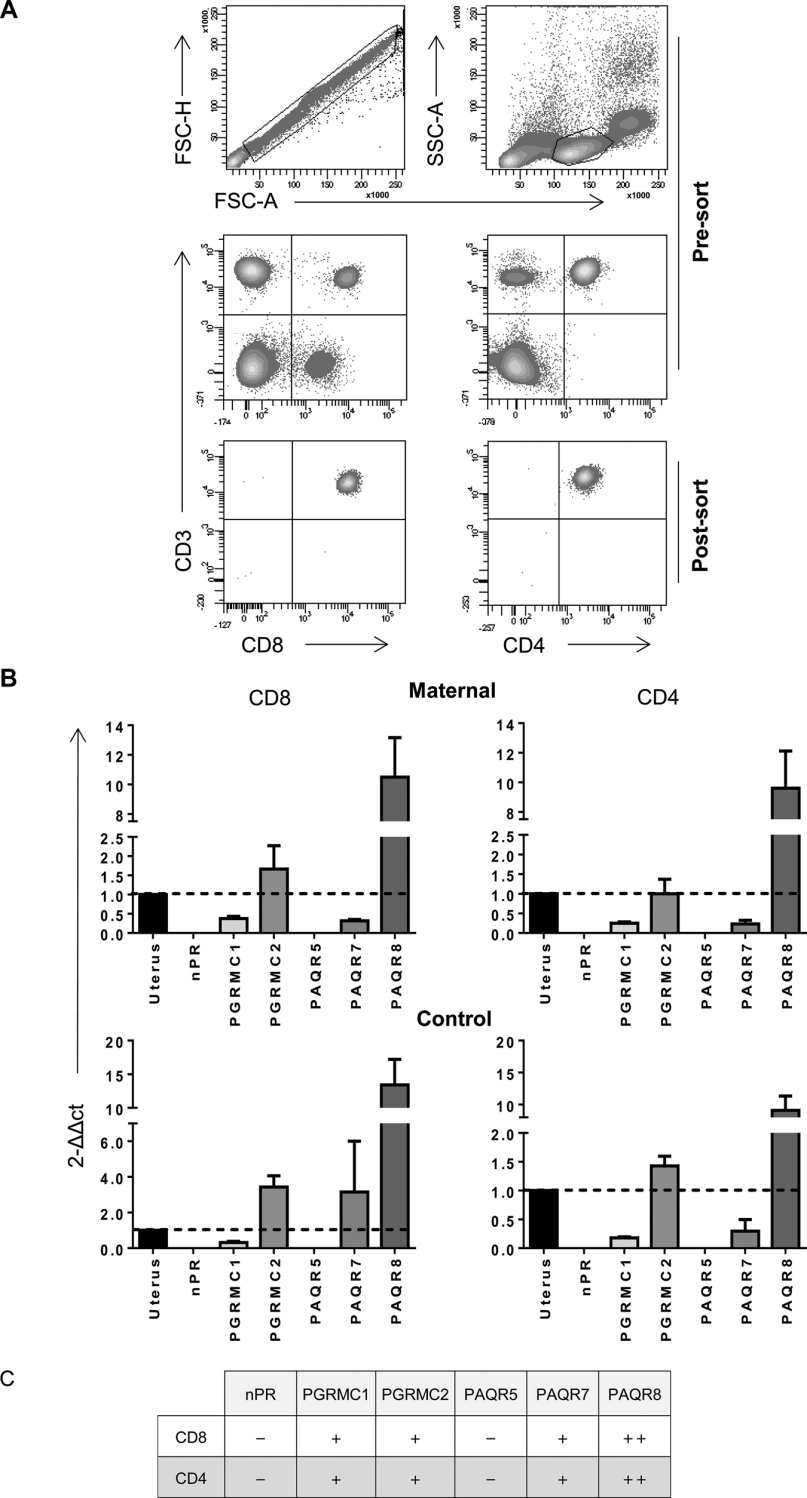
The expression of membrane and nuclear progesterone receptors on CD8^+^ and CD4^+^ T cells. (A) PBMCs isolated from maternal and control donors were sorted into pure populations of CD8^+^ and CD4^+^ T cells and purity was checked (*n* = 4). (B) RNA was extracted from the CD8^+^ and CD4^+^ T‐cell populations, and real‐time PCR was used to determine the expression of the nuclear progesterone receptors (nPR), progestin and adipoQ receptor family members 5, 7, and 8 (PAQR5, 7, and 8), and the progesterone receptor membrane component members 1 and 2 (PGRMC1 and 2). Human uterus cDNA was used as a positive control and GAPDH as the endogenous control/housekeeper. Data shown as mean + SEM of *n* = 4 samples in each group. (C) Summary of nuclear and membrane progesterone receptor expression levels found on CD8^+^ and CD4^+^ T cells relative to the levels in uterus.

Following our observation of a profound influence of progesterone on the pattern of cytokine production from primary T cells, we next examined the expression of PRs by CD4^+^ and CD8^+^ T cells. Quantitative PCR was used to examine the pattern of expression of both nuclear and membrane‐associated PRs. CD4^+^ and CD8^+^ T cells were sorted from maternal and control PBMC samples (Fig. 8A), and RNA was then extracted and used to synthesize cDNA. Uterine cDNA was used as a positive control (Fig. 8B).

No differences were observed between PR expression on T cells taken from maternal donors or controls. Importantly, nuclear PR expression was not detected on any T‐cell subset. The mPR family member PAQR5 was also not detectable. In contrast, both members of the PGRMC mPR family were detectable within T cells, as were PAQR7 and PAQR8. This suggests the effects of progesterone on CD8^+^ or CD4^+^ T cells are likely mediated through one of these four membrane receptors, whereas nuclear PRs cannot play a significant role.

## Discussion

Our study has characterized how progesterone, at concentrations similar to those found at the maternal–fetal interface, can directly induce a profound and unique influence on the functional response of T cells following stimulation. The hormone markedly alters the pattern of cytokine production by CD4^+^ and CD8^+^ T cells, strongly biasing it toward an IL‐4 profile, and also suppresses cellular proliferation. This information reveals important insights into the immunobiology of pregnancy and is likely to be directly relevant to studies of the potential mechanisms by which progesterone therapy may serve to reduce fetal loss.

The longstanding paradigm of immune responsiveness during pregnancy is that fetal tolerance is maintained through a shift in the pattern of cytokine production from a “Th1 type” profile toward more of a Th2 pattern 22. It is now recognized that this is an oversimplification of the interplay of individual cytokines during pregnancy, but it remains an important concept for the maintenance of successful pregnancy. Indeed, an abnormal skewing of maternal cytokine production toward a “Th1 type” profile is a recognized feature in pathological human pregnancies, including recurrent miscarriage and preterm labor 9, 23, 24. Our findings that treatment with 10 μM progesterone resulted in a significant reduction in the production of the pro‐inflammatory Th1 cytokines IFN‐γ and TNF‐α by both CD4^+^ and CD8^+^ T cells and a corresponding increase in the percentage of cells expressing the classical Th2 cytokine IL‐4, most notably by CD8^+^ T cells, are therefore important and in keeping with this being a mechanism by which progesterone may promote fetal tolerance. These results are also consistent with earlier studies that demonstrated a reduction in IFN‐γ and TNF‐α production, and increased levels of IL‐4, when PBMCs were treated with mitogen and progesterone 11, 25. In this study, we have been able to comprehensively characterize these changes and determine their relative influence on CD4^+^ and CD8^+^ T cells.

However, limitations of the binary Th1/Th2 paradigm are now very well described. Additional T‐cell subsets are now recognized as important 9 and T‐cell cytokine function shows far more diversity and plasticity than this schema suggests (reviewed in 24). Furthermore, the important physiological role of Th1‐type cytokines during events such as implantation and initiation of labor are well recognized 24. Our findings are also consistent with this more subtle and complex understanding of T‐cell biology during pregnancy, as we found that the percentage of cells expressing IL‐5 and IL‐10, which would classically be described as Th2 cytokines, is significantly decreased following treatment with 10 μM progesterone.

While IL‐10 was originally isolated from Th2 cells, it is now recognized to be produced by many cell types including Th1 cells 26, and placental trophoblast 27. It has a diverse range of effects including the regulation of immune responses to infection and protection of the host from tissue damage due to inflammation 28. It has also been suggested to have a role in pregnancy in promoting fetal tolerance 29, although IL‐10 knockout mice continue to have successful pregnancies 30. IL‐10 has also been found to reduce clearance of infectious organisms from the placenta 27. The cytokine therefore demonstrates functions that may be both potentially beneficial and deleterious to successful pregnancy outcome, and understanding why progesterone reduces IL‐10 production by CD4^+^ and CD8^+^ T cells at concentrations present in the human placenta will require further investigation.

The finding of progesterone increasing IL‐4 production by CD8^+^ T cells is intriguing, with expression of this cytokine normally associated with CD4^+^ T cells, although there have been previous reports of a regulatory‐like CD8^+^ T‐cell subset that expresses IL‐4 but little IFN‐γ 31, 32. It is therefore possible that shifting the profile of CD8^+^ T cells from those with a cytotoxic profile toward a less inflammatory IL‐4‐expressing subset may be important for fetal tolerance. It is also of note that maternal CD8^+^ T cells isolated from women during pregnancy were more sensitive to the action of progesterone. The mechanism behind this is uncertain but a distinct biological advantage could be envisaged from this.

Our data demonstrates clearly a marked dose–response relationship in the immunological effects of progesterone. At lower concentrations of progesterone (1 μM), levels that are comparable to those seen within maternal serum during human pregnancy, the immunological effects were present but subtle. In contrast, when the concentration of progesterone was increased to 10 μM, which is equivalent to the level found within the decidua, the effects are much more profound, with a marked suppression of IFN‐γ and TNF‐α, but an increase in IL‐4 production. This dose–response effect may ensure that at lower progesterone concentrations within the peripheral immune system, the effects on T‐cell function are modest and permit maintenance of an active immune response to infection. However, at sites nearer to the fetus, the progesterone concentrations are capable of inducing profound changes in T‐cell cytokine secretion that can facilitate successful pregnancy. The measurement of peripheral progesterone concentrations or serum cytokine levels may therefore be rather uninformative in this context, and not representative of events at the fetal–maternal interface. Indeed, peripheral cytokine changes did not appear to correlate well with pregnancy outcome or the therapeutic use of the progesterone analog dydrogesterone when this was examined in a recent clinical trial 33.

Our work shows that progesterone treatment not only changes the profile of cytokine production by CD8^+^ and CD4^+^ T cells but also reduces the number of cytokines simultaneously produced by these cells. In particular, progesterone was seen to reduce cytokine polyfunctionality while polarizing cytokine production in a dose‐dependent fashion toward combinations that include IL‐4 but exclude IFN‐γ. Polyfunctionality is regarded as an important marker of the quality of the T‐cell response, and has been correlated with the ability to provide protection from infection (reviewed in 34). In particular, T cells expressing multiple Th1 cytokines such as IFN‐γ and TNF‐α have been shown to be beneficial in several studies of infectious disease 35, 36, 37. It is therefore again likely that the highly dose‐dependent effects of progesterone on polyfunctional cytokine production reflect the need to establish a progesterone concentration gradient between the periphery and the maternal–fetal interface. Interestingly, we found that maternal T cells, when compared to controls, also demonstrated a more marked focussing and skewing of the T‐cell response when treated with progesterone, though the mechanism behind this remains unknown.

We were interested to study if the effect of progesterone on T‐cell function would be more apparent on cell clones that had been primed during natural pregnancy. We therefore isolated alloreactive CD8^+^ T‐cell clones from pregnant women that had specificity for paternal HY peptides expressed by the fetus. Incubation of these clones with progesterone did lead to an alteration in the profile of cytokine production in response to peptide stimulation, however, this was not different from the pattern that was observed with the use of T‐cell clones specific for viral peptide. These data indicate that physiological conditions present at the time of priming of an antigen‐specific T‐cell response may not play a dominant role in determining later susceptibility to the effect of exposure to progesterone. As such, the immunomodulatory effects of progesterone on T‐cell cytokine production in vivo will be mediated as a function of its concentration, rather than by the antigen specificity of the T cells concerned.

One striking feature of our work is that it supports the growing appreciation of the considerable plasticity of human T cells 38. Progesterone was able to markedly alter cytokine secretion from mature T cells, such as viral specific T‐cell clones, that naturally demonstrate a pattern of polarized cytokine production. This observation offers insights into understanding the plasticity of human T cells and has interesting implications for the use of progesterone as an agent to modulate antigen‐specific T‐cell function in vitro, and which may be of utility in the expanding field of T‐cell therapy.

A number of studies have identified that progesterone may exert its effects on T cells through rapid and reversible blockade of voltage‐gated and calcium‐activated K^+^ channels 39, 40, 41. We confirmed that the classical nuclear PRs are not expressed on either CD4^+^ or CD8^+^ T cells. However, we were able to detect four of the five known mPRs. This suggests that the effects we have characterized are also likely to be mediated through mPRs and further work will be important in determining the underlying mechanisms and elucidating why T cells taken from pregnant women are more sensitive to some progesterone effects.

**Figure 9:**
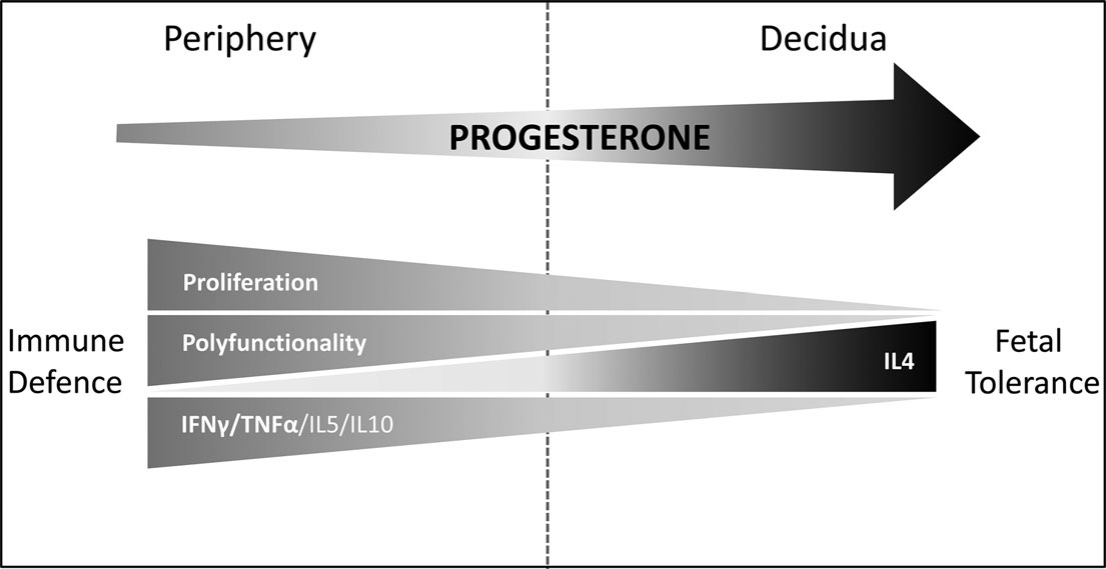
Schematic representation of the influence of progesterone concentration on CD4^+^ and CD8^+^ T‐cell function during pregnancy. Exposure of maternal T cells to progesterone at concentrations similar to those found at the maternal–fetal interface (right) induced a unique skewing of the cytokine production profile of CD4^+^ and CD8^+^ T cells, with reductions in potentially deleterious IFN‐γ and TNF‐α production but also reductions in IL‐10 and IL‐5. Conversely, production of IL‐4 was increased. Maternal T cells became less polyfunctional, with fewer cytokines produced but tending to include IL4. This was accompanied by reduced T‐cell proliferation. CD4^+^ and CD8^+^ T cells responded to progesterone in a dose‐dependent manner, with only subtle effects at concentrations comparable to those in maternal peripheral blood (left) facilitating continued host defense. Thus, the tolerogenic effects are localized to the maternal–fetal interface where higher concentrations of progesterone are found (right).

Based on our findings, we suggest a model for the effects of progesterone on T cells during pregnancy, which are summarized schematically in Figure 9. We propose that progesterone induces a unique and profound skewing of the profile of cytokine production from CD4^+^ and CD8^+^ T cells, which is irrespective of antigenic specificity. This is characterized by not only a reduction in the production of pro‐inflammatory cytokines such as IFN‐γ and TNF‐α, but also a substantial decrease in release of IL‐10 and IL‐5. The production of IL‐4 is increased, and cells become less polyfunctional with a focussing of cytokine production toward profiles that include IL‐4. These changes are associated with a coincident and marked reduction in the proliferation of cells following stimulation. As T cells respond to progesterone in a dose‐dependent manner, the progesterone concentration gradient between the maternal–fetal interface and the periphery allows maintenance of effective immune responses within peripheral tissues but provides a tolerogenic environment within the uterus.

These findings have important implications for our understanding of the normal biology of pregnancy and the role of progesterone in maintaining fetal tolerance. Progesterone therapy is used widely as a means to try to support pregnancies that are at risk of fetal loss. Indeed, there is mounting evidence from clinical trials of the efficacy of progesterone in women with recurrent miscarriage or high risk of preterm delivery 13, 14, 33. Our findings provide further information on the biological rationale behind the use of progesterone as an immunomodulatory agent in the context of these problems, to counteract the observed abnormal skewing of the maternal T‐cell response toward a Th1 phenotype in these cohorts 23, 24. Indeed, it is possible that patient stratification through measurement of immunological parameters may allow identification of those patients most likely to benefit from treatment, or facilitate the rational design of new therapeutic approaches.

## Materials and methods

### Study participants

Healthy pregnant women without previous medical or obstetric complications were recruited from antenatal clinics at Birmingham Women's National Health Service Foundation Trust, United Kingdom, between June 2011 and June 2013. The median age of the cohort was 30 years (range 23–35), with a median gestation of 90 days (12 + 6 weeks; range 75–284 days). Healthy male donors were used as controls, as they have low peripheral blood progesterone concentrations, in contrast to the pregnant cases. Health males were used rather than nonpregnant females as there are wide fluctuations in progesterone concentration during the menstrual cycle. The progesterone concentration in men (<0.6–4.45 nmol/L) is similar to that found in the follicular stage of the menstrual cycle (<0.6 nmol/L) 42. Healthy controls were volunteers from staff members at the University of Birmingham, with median age of 27 years (range 22–51). Venous blood samples between 14 and 24 mL were obtained.

The study was approved by the University of Birmingham, Human Bioresources Centre for tissue collection and use (HBRC Ref: 10–024).

### Intracellular cytokine assay

PBMCs were extracted from venous blood of healthy maternal and control donors, activated with PHA (Remel, Kent, UK), and treated with either DMSO (vehicle control, at 0.15%) or indicated progesterone concentrations for 48 h at 37°C, 5% CO_2_ in RPMI 1640 complete media (containing penicillin/streptomycin (50 μg/mL), l‐glutamine (2 mM; Life Technologies, Paisley, UK), 10% fetal calf serum (PAA, Yeovil, UK). After 44‐h incubation, monensin (Sigma‐Aldrich, Dorset, UK) was added to the cells to prevent the release of cytokines from cells. Cells were harvested, and stained with the surface phenotype antibody panel (Supporting Information Table 1) for 20 min on ice, in the dark. Cells were washed in MACS buffer (PBS/0.5% BSA/2 mM EDTA) and fixed in 2% PFA (Sigma‐Aldrich) for 30 min at room temperature, in the dark. Cells were washed in MACS buffer followed by permeabilization in 0.5% Saponin solution (Sigma‐Aldrich) for 10 min at room temperature, in the dark. Cells were stained with the intracellular antibody panel (Supporting Information Table 1) for 20 min at room temperature, in the dark before a final wash step in MACS buffer. Analysis was carried out using an LSR II flow‐cytometer (BD Biosciences, San Jose, CA, USA) and FACSDiva software (version 6.1.3).

Proliferation assays were performed as above except that prior to PHA treatment cells were labeled with CellTrace™ Violet (Life Technologies) according to the manufacturer's instructions.

### HY‐specific CD8^+^ T‐cell clone assays

HY‐specific CD8^+^ T‐cell clones were isolated as previously described 3. Cells were sampled and stained with HY‐specific dextramer (HLA*0201, FIDSYICQV; Immudex, Denmark) or with an HLA*0201‐negative control dextramer (Immudex) at room temperature for 20 min, washed in MACS buffer, and stained for CD3^+^ and CD8^+^ (Supporting Information Table 1).

Clones that were confirmed to stain dextramer were subsequently used to assess the effects of progesterone on HY‐specific T cells. HLA*0201‐positive female LCL cells were incubated with 5 mg/mL of either HY peptide (FIDSYICQV; Alta Biosciences, Birmingham, UK) or MAGEA4 peptide (GVYDGREHTV; Alta Biosciences) in serum‐free RPMI 1640 for 1–2 h. Cells were then resuspended in RPMI 1640 complete media and irradiated. Clones and irradiated LCLs were resuspended in complete media supplemented with IL‐2 (20 U/mL) and clones were mixed with either HY peptide or MAGEA4 peptide loaded LCLs in a 1 to 10 ratio. Cells were treated with either DMSO (vehicle control), or 1 or 10 μM progesterone and the assay continued as described previously for the intracellular cytokine assay.

### Separation of CD8^+^ and CD4^+^ T‐cell subsets using the Mo‐Flow cell sorter

PBMCs were surface stained with the sorting antibody panel as per Supporting Information Table I. Sorting was carried out on the Mo‐Flow Cell Sorter (Beckman Coulter, High Wycombe, UK). Following sorting, purity was assessed using the LSR II flow cytometer. The cells obtained were pelleted and stored at –20°C in RNAlater (Sigma‐Aldrich) until required.

### RNA isolation, cDNA synthesis, and quantitative real‐time PCR

RNA was extracted from sorted cells with the RNeasy Plus micro kit (Qiagen, Manchester, UK) according to the manufacturer's instructions. RNA was converted to cDNA using random primers and M‐MLV reverse transcriptase (Promega, Southampton, UK). Uterus RNA (AMSBio, Abingdon, UK) was used to generate cDNA for a positive control.

For quantitative real‐time PCR human taqman gene expression assays (Applied Biosystems (Life Technologies), Paisley, UK) were used for the PRs; PR (Hs01556702_m1), PAQR5 (Hs00214508_m1), PAQR7 (Hs00753107_s1), PAQR8 (Hs00370233_m1), PGRMC1 (Hs00998344_m1), and PGRMC2 (Hs01128672_m1). For the housekeeping gene, the GAPDH control kit was used (Yakima yellow/Eclipse, Eurogentec, Southampton, UK). DNA amplification was carried out on the ABI Prism 7500 sequence detection system. After an initial activation of uracil‐*N*‐glycosylase (2 min at 50°C and 10 min at 95°C), amplification was carried out for 40 cycles (15 s at 95°C, 60 s at 60°C).

### Statistical analysis

Statistical analysis was performed using GraphPad Prism version 5 (GraphPad software). To compare the three treatment groups, a one‐way ANOVA was used with a Bonferroni post‐hoc test. To determine differences in cytokine production between maternal and controls groups, a two‐tailed *t*‐test was used. Differences in the number of cytokines expressed were determined using a repeated measure ANOVA with a Bonferroni post‐hoc test. Differences in PR expression were analyzed using a Mann–Whitney *U* test. The null hypothesis was rejected at a *p* value of <0.05.

## Conflict of interest

The authors declare no financial or commercial conflict of interest.

**Abbreviations**
mPR: membrane progestin receptor
PAQR: progestin and adipoQ receptor
PGRMC: progesterone receptor membrane component

## Supporting information

As a service to our authors and readers, this journal provides supporting information supplied by the authors. Such materials are peer reviewed and may be re‐organized for online delivery, but are not copy‐edited or typeset. Technical support issues arising from supporting information (other than missing files) should be addressed to the authors.

Figure S1.
Figure S2.
Figure S3
Figure S4
Figure S5
Table S1: Antibodies used in this study

peer review correspondence
