# Supplementary material for: Progesterone promotes maternal–fetal tolerance by reducing human maternal T‐cell polyfunctionality and inducing a specific cytokine profile
Source: Eur J Immunol. 2015 Aug 28;45(10):2858–72. doi: 10.1002/eji.201445404 (PMC4833190; doi:10.1002/eji.201445404)
Supplement: Supplementary file 1 — Figure S1. Figure S2. Figure S3 Figure S4 Figure S5 Table S1: Antibodies used in this study [file EJI-45-2858-s001.pdf]

European Journal of Immunology

Supporting Information for

DOI 10.1002/eji.201445404

David Lissauer, Suzy A. Eldershaw, Charlotte F. Inman, Aravinthan Coomarasamy, Paul A. H. Moss and Mark D. Kilby

Progesterone promotes maternal–fetal tolerance by reducing human maternal T-cell polyfunctionality and inducing a specific cytokine profile

© 2015 The Authors. *European Journal of Immunology* published by WILEY-VCH Verlag GmbH & Co. KGaA, Weinheim.

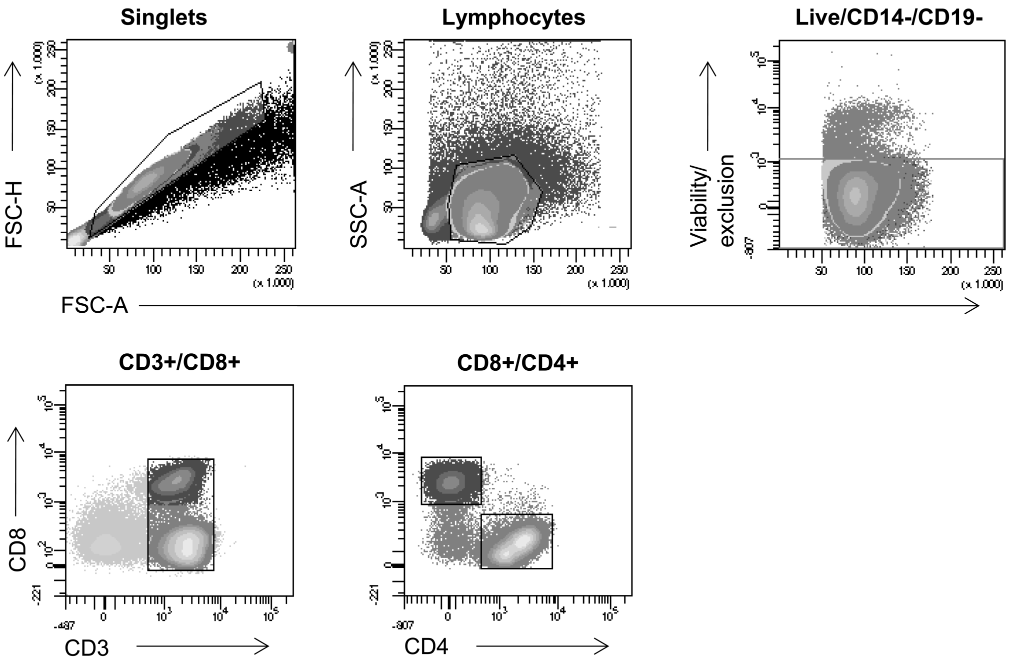

Supporting information Figure 1. Flowcytometry gating strategy. FSC-H and FSC-A were used to identify singlets. Lymphocytes are then gated using FSC-A versus SSC-A. Dead cells, CD14+ and CD19+ cells are excluded using viability/exclusion channel before CD3+ cells are selected and then CD4+ and CD8+ cells are gated to look at cytokine expression.

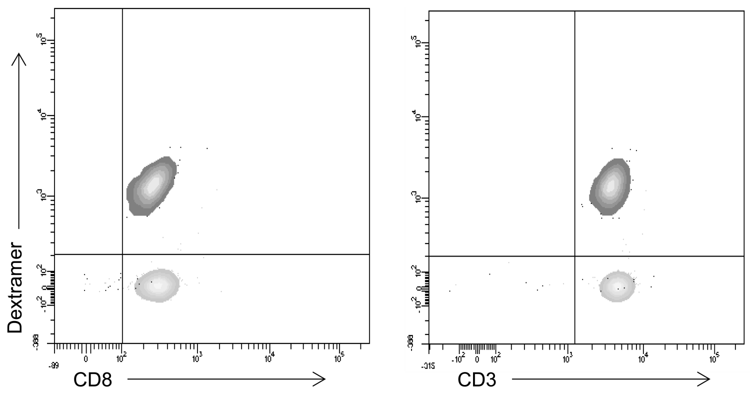

Supporting information Figure 2. Antigen specificity of CD8+ T cell clones. Flow cytometry plots illustrate the antigen specificity of CD8+ T cell clones, using MHC-peptide dextramer specific for HY antigen (HLA*0201 FIDSYICQV PE, shown in black) compared to negative dextramer (HLA*0201 neg PE, shown in grey).

LSRII configuration	Laser	Filter	Antibody	Clone	Conjugate
Surface stain	488	610/20	Viability Dye[a]		
			CD14[b]	RMO52	ECD
			CD19[b]	J3-119	ECD
		695/40	CD4[c]	RPA-T4	PerCP-Cy5.5
	405	525/50	CD8[d]	RPA-T8	V500
	633	780/60	CD3[e]	HIT3a	APC-Cy7
Intracellular stain	488	530/30	IL5[f]	9906	FITC
		575/26	IL10[c]	JES3-9D7	PE
		780/60	TNFα[c]	Mab11	PE-Cy7
	405	450/50	IL17[e]	BL168	PB
	633	660/20	IL4[e]	8D4-8	APC
		730/45	IFNγ[e]	4S.B3	AF700
Clone specificity	488	575/26	Dextramer[g]		PE
		610/620	Viability Dye[a]		
	405	525/50	CD8[d]	RPA-T8	V500
	633	780/60	CD3[e]	HIT3a	APC-Cy7
Proliferation	405	450/50	CellTrace Violet[a]		

Supporting information Table I. LSR II configurations and reagents used

a=Lifetechnologies, b=Beckman Coulter, c= Ebiosciences, d= BD biosciences, e= Biolegend, f= R&D systems, g= Immunodex
