# Supplementary material for: Progesterone promotes maternal–fetal tolerance by reducing human maternal T‐cell polyfunctionality and inducing a specific cytokine profile
Source: Eur J Immunol. 2015 Aug 28;45(10):2858–72. doi: 10.1002/eji.201445404 (PMC4833190; doi:10.1002/eji.201445404)
Supplement: Supplementary file 2 — peer review correspondence [file EJI-45-2858-s002.pdf]

European Journal of Immunology

# Peer review correspondence

**Progesterone promotes maternal-fetal tolerance by reducing human maternal T-cell polyfunctionality and inducing a specific cytokine profile**

D. Lissauer, S.A. Eldershaw, C.F. Inman, A. Coomarasamy, P.A.H. Moss and M.D. Kilby

Corresponding author: David Lissauer, Academic department, Birmingham Women's Hospital, School of Clinical and Experimental Medicine, College of Medical and Dental sciences, University of Birmingham, United Kingdom

---



| | | |
|---|---|---|
| Review Timeline: | Submission date: | 11 December 2014 |
| | Editorial decision: | 13 March 2015 |
| | Revision received: | 19 May 2015 |
| | Accepted: | 30 July 2015 |



---

Handling Executive Committee member: Prof. Annette Oxenius

Please note that the correspondence below does not include the standard editorial instructions regarding preparation and submission of revised manuscripts, only the scientific revisions requested and addressed.

First Editorial Decision – 13 March 2015

Dear Dr. Lissauer,

Thank you for your prolonged patience while we evaluated the peer review of your manuscript ID eji.201445404 entitled "Progesterone modulates human maternal CD4+ and CD8+ T cell function, reducing polyfunctionality and inducing a specific cytokine profile to promote maternal-fetal tolerance" which you submitted to the European Journal of Immunology. As you know, we initially felt we needed more information regarding your choice of control donors, and subsequently we asked for more detailed comments from referee 2. All opinions have now been received and the comments of the referees are included at the bottom of this letter.

A revised version of your manuscript that takes into account the comments of the referees will be reconsidered for publication.

European Journal of Immunology

You should also pay close attention to the editorial comments included below. *In particular, please edit your figure legends to follow Journal standards as outlined in the editorial comments. Failure to do this will result in delays in the re-review process.*

Please note that submitting a revision of your manuscript does not guarantee eventual acceptance, and that your revision will be re-reviewed by the referees before a decision is rendered.

If the revision of the paper is expected to take more than three months, please inform the editorial office. Revisions taking longer than six months may be assessed by new referees to ensure the relevance and timeliness of the data.

Once again, thank you for submitting your manuscript to European Journal of Immunology and we look forward to receiving your revision.

Yours sincerely,
Karen Chu

On behalf of Prof. Annette Oxenius

Dr. Karen Chu
Editorial Office
European Journal of Immunology
e-mail: ejied@wiley.com
www.eji-journal.eu

******************************************

Reviewer: 1
Comments to the Author
In their study “Progesterone modulates human maternal CD4+ and CD8+ T cell function, reducing polyfunctionality and inducing a specific cytokine profile to promote maternal-fetal tolerance” Lissauer and colleagues investigated dose-dependent effects of progesterone on human CD4+ and CD8+ T cell function mainly focusing on cytokine production and proliferation of the two T cell subsets. An influence of progesterone on T cell behavior was already addressed by several other research groups. However, the study presented by Lissauer and colleagues provide a detailed analysis of cytokine secretion patterns of CD4+ and CD8+ T cells in the presence of progesterone. This also includes the analysis of the polyfunctionality of these cells. Interestingly, the authors found that the effects mediated by progesterone

European Journal of Immunology

are rather dose dependent than antigen dependent. Altogether, the manuscript is well structured and well written. The reviewer has some minor points for the authors.

Minor concerns

1) General: In the material and methods section the authors stated why they chose male samples as controls. However, the reviewer feels that healthy non-pregnant woman would be the better choice as controls. It is well known that immune responses differ between females and males and it cannot be excluded that PBMCs/T cells taken for the analysis were already pre-primed. Thus, for future studies the reviewer would recommend to take samples from non-pregnant woman being in the same cycle stage as controls.
2) Abstract: The authors stated that nuclear P4 receptors can be found in murine T cells. However, this seems not to be the case for human T cells. To underline the difference between the two species the reviewer would recommend to add the word “human” in front of “ T cells” (page 2, line 29-30)
3) Introduction: On page 3, line 47 the authors provided P4 concentrations in brackets. The reviewer assumes that <1µM 3rd trimester refers to the concentration in the serum? Is this correct? This point should be clarified.
4) Methods: The authors stated that samples were taken from gestation day 75 until 284. This includes all three trimester. How can the authors be sure that T cells from the third trimester behave the same way as T cells from the first trimester? They are pre-primed by different doses of P4.
5) Results: In Figure 1, cytokine production was measured in PBMCs isolated from healthy maternal and control donors. The reviewer supposes that “healthy maternal donors” are pregnant women? and “control donors” are males? Later on, in Figure 3, the authors talk about “pregnant women” and in Figure 4 maternal and males samples are compared. Using different terms for the same sample material “healthy maternal/pregnant/maternal” or “control/males” irritates the reader. Thus, the reviewer would recommend using one term consistently throughout the manuscript.
6) Results: On page 12, lines 50 and 52 the authors refer to Figure 1 instead of Figure 8.

Reviewer: 2
Comments to the Author - original comments
Lissauer et al are describing studies of human T cells assessing the consequences of progesterone for T cell function. The overall conclusions are that progesterone treatment enhances IL-4 production while reducing overall T cell polyfunctionality. While these data are of interest, there are several important limitations, including:
- novelty: as also stated by the authors in the discussion, a large part of the presented data confirm previous findings

European Journal of Immunology

- focus on in vitro studies: it remains unclear how much the in vitro incubation of T cells with progesterone at different levels reflects in vivo situations
- the data on receptor expression and their relevance are very limited, and the paper therefore remains very descriptive, lacking any mechanistic studies.

Comments to the Author - further comments
One concern remains novelty, as highlighted in my initial review, and the authors should address this.

In terms of additional experiments, it would be important to understand how progesterone levels might modulate T cell function. The simple description of potential receptor expression profiles is not providing a functional link. One could use T cell clones to knock out these receptors. The authors might think that this is beyond the scope of the paper, but without such data the paper only shows some in vitro effect of progesterone on T cell function, what is not very novel.

Reviewer: 3
Comments to the Author
This study shows that progesterone - in a concentration-dependent fashion- influences cytokine production and proliferation of CD4 and CD8+ cells from pregnant women as well as those from males. While T cell cytokine production skews towards Th2, while the cells become less polyfunctional. The effect of progesterone seems to be not antigen-specific.
The study is well designed and the data are interesting.

-Fig. 1 shows that the frequency of cytokine expressing cells (with the only exception of IL-4 in CD8+cells) is lower in pregnant women's than in male lymphocytes. On Fig. 4 there is not much difference between the effect of progesterone on pregnancy and male lymphocytes, furthermore, the expression pattern of progesterone receptors also seem to be similar both in CD4+ and CD8+ lymphocytes from the two groups. What is the reason for the higher sensitivity of CD8+ pregnancy lymphocytes to progesterone? How can it be explained that progesterone acts solely on IL-4 production, which makes all the difference in the maternal immune function?
-Why were the control cells (without progesterone) treated with DMSO?

European Journal of Immunology

First revision – authors' response – 19 May 2015

Reviewer: 1

Comments to the authors

In their study "Progesterone modulates human maternal CD4+ and CD8+ T cell function, reducing polyfunctionality and inducing a specific cytokine profile to promote maternal-fetal tolerance" Lissauer and colleagues investigated dose-dependent effects of progesterone on human CD4+ and CD8+ T cell function mainly focusing on cytokine production and proliferation of the two T cell subsets. An influence of progesterone on T cell behavior was already addressed by several other research groups. However, the study presented by Lissauer and colleagues provide a detailed analysis of cytokine secretion patterns of CD4+ and CD8+ T cells in the presence of progesterone. This also includes the analysis of the polyfunctionality of these cells. Interestingly, the authors found that the effects mediated by progesterone are rather dose dependent than antigen dependent.

Altogether, the manuscript is well structured and well written.

Thank you for your kind comments on the manuscript.

The reviewer has some minor points for the authors.

1) General: In the material and methods section the authors stated why they chose male samples as controls. However, the reviewer feels that healthy non-pregnant woman would be the better choice as controls. It is well known that immune responses differ between females and males and it cannot be excluded that PBMCs/T cells taken for the analysis were already pre-primed. Thus, for future studies the reviewer would recommend to take samples from non-pregnant woman being in the same cycle stage as controls.

Thank you for raising this point. I previously had the opportunity to correspond with Dr Chu regarding this key issue and we recognise the challenge associated with the choice of an optimal control group for this work.

Peripheral progesterone concentration is used for numerous clinical applications and therefore has well defined reference ranges [1-3]. The challenge with using female non-pregnant controls is that in addition to changes during pregnancy there are very marked differences measured in progesterone concentrations in peripheral blood during the female menstrual cycle [2]. In men the reference range is <0.6-4.45 nmol/L. In women during the follicular phase of the menstrual cycle the concentration is <0.6 nmol/L, increasing to 9.54-63.6 nmol/L during the luteal phase [1]. In pregnancy peripheral blood progesterone concentrations increase throughout gestation, from 25.4-152.6 nmol/L during the 1st trimester to 314.8-1087.5 nmol/L during the 3rd trimester[3].

For this study our aim was to select as the control group those with a low peripheral blood progesterone concentration to demonstrate most clearly any differential immunomodulatory effects of progesterone treatment. Hence the choice to contrast the cases of pregnant women, with males controls who have low progesterone levels. Progesterone concentrations in male donors are equivalent to women in the follicular phase of the menstrual cycle. The use of male controls rather than female controls sampled during the follicular phase of their cycle also offered critical practical advantages. It meant that we did not require a detailed menstrual, contraceptive and pregnancy history to be obtained from the control laboratory donors, which would otherwise have been needed. Furthermore, the assays were conducted using freshly isolated peripheral blood mononuclear cells and the use of male controls rather than female controls during the follicular phase of their menstrual cycle meant that controls with freshly isolated peripheral blood mononuclear cells could be run with every assay.

Other studies, already cited in the manuscript, which have similarly studied in vitro the effect of the addition of progesterone on T cell function have also used lymphocytes from males to avoid the variability in natural progesterone levels if using cells from females [4, 5].

We recognize there would be important scientific merit in extending our studies as suggested by the reviewer to include further groups such as contrasting the effects in non-pregnant women between the follicular and luteal phases of the menstrual cycle.

We have added further clarification to address this in the manuscript by expanding the material and methods section to state:

Healthy male donors were used as controls, as they have low peripheral blood progesterone concentrations, in contrast to the pregnant cases. Health males were used rather than non-pregnant females as there are wide fluctuations in progesterone concentration during the menstrual cycle. The progesterone concentration in men (<0.6 – 4.45 nmol/L) is similar to that found in the follicular stage of the menstrual cycle (<0.6 nmol/L) [51].

2) Abstract: The authors stated that nuclear P4 receptors can be found in murine T cells. However, this seems not to be the case for human T cells. To underline the difference between the two species the reviewer would recommend to add the word “human” in front of “ T cells” (page 2, line 29-30)

Thank you, we have made this change as recommended.

3) Introduction: On page 3, line 47 the authors provided P4 concentrations in brackets. The reviewer assumes that <1µM 3rd trimester refers to the concentration in the serum? Is this correct? This point should be clarified.

We have added the clarification that this is referring to the serum concentration as suggested.

European Journal of Immunology

4) Methods: The authors stated that samples were taken from gestation day 75 until 284. This includes all three trimester. How can the authors be sure that T cells from the third trimester behave the same way as T cells from the first trimester? They are pre-primed by different doses of P4.

The reviewer raises an important point that there may be gestation specific effects of progesterone that are not explored in our study. We did conduct a secondary analysis by gestation (looking for correlation with gestational age as a continuous variable and following categorisation by trimester) and did not with this dataset reveal any statistically significant gestation specific effects. However, this type of secondary analysis is we feel susceptible to type II error given the size of the sample, and we therefore did not separately report this analysis in this manuscript. Rather we feel that this question is of sufficient merit that a future study would be warranted to address this question fully. This would be required to be adequately powered, with the benefit of knowing the expected effect sizes from our current study and with a sampling strategy to ensure adequate coverage over the full range of gestational ages.

5) Results: In Figure 1, cytokine production was measured in PBMCs isolated from healthy maternal and control donors. The reviewer supposes that “healthy maternal donors” are pregnant women? and “control donors” are males? Later on, in Figure 3, the authors talk about “pregnant women” and in Figure 4 maternal and males samples are compared. Using different terms for the same sample material “healthy maternal/pregnant/maternal” or “control/males” irritates the reader. Thus, the reviewer would recommend using one term consistently throughout the manuscript.

Thank you for highlighting this inconsistency. This has now been rectified and throughout the manuscript the terms “maternal” and “control” have now been used consistently.

6) Results: On page 12, lines 50 and 52 the authors refer to Figure 1 instead of Figure 8.

Thank you, this error has been corrected.

Reviewer: 2

Comments to the Author - original comments

Lissauer et al are describing studies of human T cells assessing the consequences of progesterone for T cell function. The overall conclusions are that progesterone treatment enhances IL-4 production while reducing overall T cell polyfunctionality. While these data are of interest, there are several important limitations, including:

- novelty: as also stated by the authors in the discussion, a large part of the presented data confirm previous findings

We would like to thank the reviewed for the recognition of the interest of our data.

The main concern of this reviewer is the novelty of our findings. We appreciate there is an existing body of work that has previously investigated the potential role of progesterone in immunomodulation during pregnancy, and as the reviewer highlighted we attempted to clearly describe this prior knowledge to

provide the reader with a clear context. But this previous work in relation to effects on T-cells was neither consistent in its conclusions nor detailed in its description of the effects. We are confident that the data we present is both novel and significant in both its contribution to our understanding of the immunology of pregnancy and because of its potential future implications for clinical practice.
Some of the main findings from our work, (as per the abstract) are shown below with the areas of novelty highlighted in italics to provide clarity on how this advances on the existing knowledge in the field:

1) A unique skewing of the cytokine production profile of CD4+ and CD8+ T-cells, with reductions in potentially deleterious IFN□ and TNF□ production but also reductions in IL10 and IL5. Conversely, production of IL4 was increased. (This profile has not been previously described, nor the respective effects on CD4 and CD8 T-cells examined separately and contrasted. Similarly a careful comparison between the effects on maternal and control T-cells has not been made.)
2) T-cells also became less polyfunctional, focussing cytokine production towards profiles including IL4. This was accompanied by reduced T-cell proliferation. (The detailed examination of the effects of progesterone on T-cell polyfunctionality and the constituent cytokines have never been previously reported).
3) Using fetal and viral antigen-specific CD8+ T-cell clones, we confirmed this as a direct, non-antigen-specific effect. (The effect of progesterone on fetal antigen specific T-cell clones has never before been examined, nor the comparison made with viral specific T-cell clones or progesterone's effects on T-cells responding to their natural cognate antigens observed.)
4) Yet human T-cells lacked conventional nuclear progesterone receptors, implicating a membrane progesterone receptor. (This has previously been a controversial subject with conflicting findings in the literature and differences between human and murine T cells).
5) CD4+ and CD8+ T-cells responded to progesterone in a dose-dependent manner, with subtle effects at concentrations comparable to those in maternal blood, but profound effects at concentrations similar to those at the maternal-fetal interface. (The use of physiological concentrations, unlike many of the previous studies, makes this much more relevant to in-vivo effects and of relevance to clinicians. The clear demonstration of the differing effects between the serum and decidual concentrations of progesterone also has important lessons for how progesterone may act in vivo)

- focus on in vitro studies: it remains unclear how much the in vitro incubation of T cells with progesterone at different levels reflects in vivo situations

The reviewer highlights a limitation that the in vitro effects of progesterone may not fully reflect the situation in vivo. We appreciate that whilst our characterisation of the effects of progesterone were comprehensive they were indeed limited to in vitro conditions. We attempted to make these investigations as relevant as possible to the effect in nature by using natural P4 for our studies and ensuring the concentrations used reflected physiological concentrations. We acknowledge that future in vivo studies on

European Journal of Immunology

a cohort of women exposed to therapeutic concentrations of progesterone supplementation or placebo would be valuable in determining if the effects we have described are also consistently seen with progesterone supplementation.

- the data on receptor expression and their relevance are very limited, and the paper therefore remains very descriptive, lacking any mechanistic studies. In terms of additional experiments, it would be important to understand how progesterone levels might modulate T cell function. The simple description of potential receptor expression profiles is not providing a functional link. One could use T cell clones to knock out these receptors. The authors might think that this is beyond the scope of the paper, but without such data the paper only shows some in vitro effect of progesterone on T cell function, what is not very novel.

We defined the nature of progesterone receptor gene expression on maternal and control T cells but appreciate the point that future studies will be important to identify which of the membrane receptors are specifically responsible. However, knocking out these multiple receptors simultaneously in primary T cells offers very considerable technical challenges and appropriate receptor specific blockers are not available. We acknowledge this limitation in the discussion of the manuscript stating “We confirmed that the classical nuclear progesterone receptors are not expressed ….. However, we were able to detect four out of the five known membrane progesterone receptors. …… further work will be important in determining the underlying mechanisms “. We feel that despite this limitation there are important and novel finding of our work as described above that warrant its publication.

Reviewer: 3
Comments to the Author
This study shows that progesterone - in a concentration-dependent fashion- influences cytokine production and proliferation of CD4 and CD8+ cells from pregnant women as well as those from males. While T cell cytokine production skews towards Th2, while the cells become less polyfunctional. The effect of progesterone seems to be not antigen-specific.
The study is well designed and the data are interesting.

Thank you for you kind comments on the design and interest of the study.

-Fig. 1 shows that the frequency of cytokine expressing cells (with the only exception of IL-4 in CD8+cells) is lower in pregnant women’s than in male lymphocytes. On Fig. 4 there is not much difference between the effect of progesterone on pregnancy and male lymphocytes, furthermore, the expression pattern of progesterone receptors also seem to be similar both in CD4+ and CD8+ lymphocytes from the two groups.
Figure 1 was an initial set of experiments to gauge the dose response effects of progesterone. This demonstrated 2 important findings. The first was a clear illustration of the dose response effect, including

over the physiological range of progesterone. The second suggestion from this data was that there may be some differences between maternal and control cells. However, this required further assessment and Figure 4 demonstrates the summary data from a larger cohort of donors (maternal n=13, control n=11) which indicated, as the reviewer states, the only statistically significant differences seen were that there was a higher frequency of maternal cells producing IL4 compared to controls.

What is the reason for the higher sensitivity of CD8+ pregnancy lymphocytes to progesterone? How can it be explained that progesterone acts solely on IL-4 production, which makes all the difference in the maternal immune function?

Both CD4 and CD8 T-cells demonstrated similar patterns of modulation by progesterone (Figure 2 and Figure 3). Indeed, these do not show only an action on IL4 production but significant reductions in TNF□, IFN□, IL10 and IL5 production, and a significant increase in IL4 production. However, whilst the increase in IL4 production in response to progesterone is seen in both CD4 and CD8 T-cells, and in both maternal and control samples, it was significantly higher in maternal CD8 T cells compared to control CD8 T cells (Figure 4).
As we acknowledge in the discussion the mechanism behind this differential action between maternal and control T cell is not yet known, but was shown not to be related to differential progesterone receptor gene expression or antigen specificity of the T-cells.

-Why were the control cells (without progesterone) treated with DMSO?

Throughout our experiments the progesterone was dissolved in DMSO. All control samples were therefore treated with an identical concentration of DMSO to the progesterone treated cells.

1 Kratz, A., Ferraro, M., Sluss, P. M. and Lewandrowski, K. B., Case records of the Massachusetts General Hospital. Weekly clinicopathological exercises. Laboratory reference values. N Engl J Med 2004. 351: 1548-1563.
2 Stricker, R., Eberhart, R., Chevailler, M. C., Quinn, F. A., Bischof, P. and Stricker, R., Establishment of detailed reference values for luteinizing hormone, follicle stimulating hormone, estradiol, and progesterone during different phases of the menstrual cycle on the Abbott ARCHITECT analyzer. Clin Chem Lab Med 2006. 44: 883-887.
3 Abbassi-Ghanavati, M., Greer, L. G. and Cunningham, F. G., Pregnancy and laboratory studies: a reference table for clinicians. Obstet Gynecol 2009. 114: 1326-1331.
4 Lai, J. N., Wang, O. Y., Lin, V. H., Liao, C. F., Tarng, D. C. and Chien, E. J., The non-genomic rapid acidification in peripheral T cells by progesterone depends on intracellular calcium increase and not on Na+/H+-exchange inhibition. Steroids 2012. 77: 1017-1024.

European Journal of Immunology

5 Chien, E. J., Liao, C. F., Chang, C. P., Pu, H. F., Lu, L. M., Shie, M. C., Hsieh, D. J. and Hsu, M. T., The non-genomic effects on Na+/H+-exchange 1 by progesterone and 20alpha-hydroxyprogesterone in human T cells. J Cell Physiol 2007. 211: 544-550.

Second Editorial Decision – 30 June 2015

Dear Dr. Lissauer,

Thank you for your patience during the delay in processing the re-review of your manuscript - there was a delay in receiving one of the reports and thereafter consultation with the Executive Editor. I have, however, good news:

It is a pleasure to provisionally accept your manuscript entitled "Progesterone modulates human maternal CD4+ and CD8+ T cell function, reducing polyfunctionality and inducing a specific cytokine profile to promote maternal-fetal tolerance" for publication in the European Journal of Immunology. For final acceptance, please follow the instructions below and return the requested items as soon as possible as we cannot process your manuscript further until all items listed below are dealt with.

Please note that EJI articles are now published online a few days after final acceptance (see Accepted Articles: http://onlinelibrary.wiley.com/journal/10.1002/(ISSN)1521-4141/accepted). The files used for the Accepted Articles are the final files and information supplied by you in Manuscript Central. You should therefore check that all the information (including author names) is correct as changes will NOT be permitted until the proofs stage.

We look forward to hearing from you and thank you for submitting your manuscript to the European Journal of Immunology.

Yours sincerely,
Karen Chu

on behalf of Prof. Annette Oxenius

Dr. Karen Chu
Editorial Office
European Journal of Immunology
e-mail: ejied@wiley.com
www.eji-journal.eu
